# The influence of perceived stress and musculoskeletal pain on work performance and work ability in Swedish health care workers

**DOI:** 10.1007/s00420-013-0875-8

**Published:** 2013-04-23

**Authors:** A. Lindegård, P. Larsman, E. Hadzibajramovic, G. Ahlborg

**Affiliations:** 1 Institute of Stress Medicine, 413 19 Göteborg, Sweden; 2 Department of Public Health and Community Medicine, Sahlgrenska Academy, University of Gothenburg, Göteborg, Sweden

**Keywords:** Stress, Musculoskeletal pain, Work ability, Work performance, Productivity

## Abstract

**Objectives:**

To evaluate the influence of perceived stress and musculoskeletal ache/pain, separately and in combination, at baseline, on self-rated work ability and work performance at two-year follow-up.

**Methods:**

Survey data were collected with a 2-year interval. Health care workers participating at both waves were included. Inclusion criteria were good self-reported work ability and unchanged self-rated work performance at baseline, resulting in 770 participants; 617 women and 153 men. Musculoskeletal pain was assessed using the question “How often do you experience pain in joints and muscles, including the neck and low back?”, perceived stress with a modified version of a single item from the QPS-Nordic questionnaire, work performance by the question “Have your work performance changed during the preceding 12 months?” and work ability by a single item from the work ability index. Associations between baseline data and the two outcomes at follow-up were analysed by means of the log binomial model and expressed as risk ratios (RR) with 95 % confidence intervals (CI).

**Results:**

A combination of frequent musculoskeletal pain and perceived stress constituted the highest risk for reporting decreased work performance (RR 1.7; CI 1.28–2.32) and reduced work ability (RR 1.7; CI 1.27–2.30) at follow-up. Separately, frequent pain, but not stress, was clearly associated with both outcomes.

**Conclusion:**

The results imply that proactive workplace interventions in order to maintain high work performance and good work ability should include measures to promote musculoskeletal well-being for the employees and measures, both individual and organizational, to minimize the risk of persistent stress reactions.

## Background

Stress-related mental disorders and musculoskeletal disorders are the two most important factors behind long-term sick leave in Sweden and account for a considerable amount of the total economic burden on society, companies and organizations (Statistics Sweden 2010). Regarding human service organizations in Sweden, structural changes during the 1990s led to a decrease in the total number of employees from 1.6 million in 1992 to 1.3 million in 2001 (Statistics Sweden 2008). This influenced not only the governing of human service organizations, but also daily tasks and performances within the organizations (Hertting et al. 2004). Along with the decrease in the number of employees, long-term sick leave due to mental disorders started to increase, and psychosocial stress at work was identified as a predominant factor behind this increase (Stefansson 2006). This rise in sick leave continued until 2003. Since then, the total amount of sick leave has gone down considerably, but still both mental disorders and musculoskeletal disorders constitutes a major reason for long-term sick leave and productivity loss within the Swedish workforce (Statistics Sweden 2011). Results from previously conducted studies have also indicated that these disorders are especially common among women working in human service organizations (Leijon et al. 2004; Fronteira and Ferrinho 2011).

Several studies have shown that reduced working capacity is a predictor of long-lasting sickness, absence and that persons at risk often scored high on instruments measuring different aspects of work-related stress (Ahola et al. 2008; Borritz et al. 2010). Moreover, it is well known that loss in productivity caused by a decreased working capacity due to medical conditions increases the so-called “hidden costs” among companies and organizations both in the long- and short-time perspectives (Stewart et al. 2003b). Thus, it is therefore of vital importance to investigate antecedents of decreased work performance and work ability in order to implement preventive strategies.

The term work performance could be defined as a combination of both quantitative and qualitative aspects of performing a work task by a worker or a work group. To objectively measure these dimensions of work are difficult, hence, most studies in this field use self-reports (de Vries et al. 2012; Waghorn and Chant 2011). Productivity on the other hand might be defined as the economic consequences of the work performance from an individual or a work group and should consequently be measured by some kind of economic measurements. However, in the scientific literature relating health to work performance and productivity, these are sometimes treated as synonymous concepts, and thus, self-reports are also frequently used to measure productivity (Brouwer et al. 1999; Hagberg et al. 2007; Martimo et al. 2010). Work performance and work productivity, as well as their potential associations and antecedents have previously been addressed in the literature. For instance, one study among computer users with musculoskeletal symptoms found a reduction in productivity by approximately 15 % for women and 13 % for men (Hagberg et al. 2002). Another study among trade firm employees showed a reduction in productivity both before and after a sick leave period by 25 and 20 %, respectively (Brouwer et al. 2002). With respect to adverse psychosocial conditions, results from previous studies suggest that high job strain is associated with decreased work performance and productivity loss (Hagberg et al. 2007; Martimo et al. 2009). Regarding the impact of mental disorders on work performance and productivity, results from a large cohort study in the US workforce have indicated a close relationship between clinical depression and productivity loss (Stewart et al. 2003a). Also, sleep disturbances, pain and negative perceptions regarding the influence of pain on work have been found to be associated with these outcomes (Hagberg et al. 2007; Martimo et al. 2010).

The concept work ability can be defined as the result of the interaction of the worker and his/her work (Ilmarinen 2004). Work ability could also be described as the balance of the workers’ resources and the work demands in terms of how well the worker at present and in the near future, is able to perform his/her work with respect to the work demands and his/her health and mental resources (Ilmarinen 2004). Work ability is, according to a large European study, strongly associated with both physical and mental well-being (Radkiewics 2005). Several risk factors for reduced work ability have previously been identified, and in a recent review, both work-related factors like high mental work demands, poor physical work environment and lack of autonomy, and individual factors like poor musculoskeletal capacity, older age and lack of leisure time physical activity were found to be associated with poor work ability (van den Berg et al. 2009).

Hence, since both musculoskeletal pain conditions and mental disorders have been proposed to be major risk factors for reduced productivity, work ability and work performance in cross-sectional studies (Stewart et al. 2003a, 2003c). It is important to investigate, especially in a study with longitudinal design, whether these factors separately or in combination constitute a risk for reduced work performance and decreased work ability among work groups with high prevalence of both the above mentioned health outcomes.

The aim of this study was thus to evaluate the influence of perceived long-lasting stress and musculoskeletal ache/pain at baseline, as well as different combinations of these potential risk factors, on self-rated reduced work ability and decreased work performance 2 years later in a group of workers exposed to a high prevalence of both musculoskeletal pain and stress.

## Methods

### Study design

This study used data from an ongoing longitudinal cohort study, aiming to investigate various psychosocial factors, perceived stress and general health among employees in two human service organizations in the south-west part of Sweden. Data were collected by means of postal questionnaires with 2-year intervals. For this, here, study data from the 2008 and 2010 questionnaires for one of the organizations, a health care organization, were used. The study was approved by the regional ethical review board in Gothenburg, Sweden and conducted according to the 1964 Declaration of Helsinki.

### Study population

The present study was based on a subsample from one of the organizations in the above mentioned population which included all health care workers (nurses, assistant nurses and physicians being the largest professional groups) participating at both waves 2008 and 2010.

At baseline, (2008) 4,739 persons in the organization were approached, and 3,481 answered the questionnaire, thus, the response rate was 73 %. At the follow-up, two years later, 292 were no longer working in the organization or had moved from the region; hence, the remaining 3,209 were approached, and the response rate was now 70 % (*n* = 2,223). The inclusion criteria were good self-reported work ability and unchanged self-rated work performance at the time for the baseline questionnaire (2008) and 12 months prior to the baseline measurements, resulting in 770 participants; 617 women and 153 men. The final study sample included only participants with complete data for all the variables used in the analyses (for outcome work ability *n* = 729, and for outcome work performance *n* = 746). There were no differences in age, gender and educational level between participants with complete data and participants excluded due to missing data.

### Assessment methods

#### Musculoskeletal pain

To assess the frequency of *musculoskeletal pain* at baseline, a single question was used; “How often do you experience pain in joints and muscles, including the neck and low back?” There were five fixed response alternatives: (a) “never”, (b) “a couple of days per month”, (c) “one day per week”, (d) “a couple of days per week” and (e) “every day”. Responses belonging to categories a, b and c were classified as “no or infrequent pain” and responses d and e were classified as “frequent pain”. This specific question has not been validated, however, it has recently been demonstrated that similar questions, that is, simple neck pain survey questions in epidemiological studies do capture features of pain with respect to health outcomes including self-reported work performance (Grimby-Ekman and Hagberg 2012).

#### Perceived stress

In order to assess the *stress* dimension at baseline, a modified version of the validated single item from the QPS-Nordic questionnaire (Elo et al. 2003) was used. The modification pertained to the time frame of perceived stress since we wanted to capture the effects of a more long-lasting stress exposure than “stress at the moment” which was the wording in the original question. The question was formulated as follows “Stress means a situation in which a person feels tense, restless, nervous or anxious or is unable to sleep at night because his/her mind is troubled all the time. Have you felt such stress during a consecutive period of at least 1 month during the preceding 12 months?” The response alternatives for this question were either “yes” or “no”. Responses belonging to the “yes” category were classified as exposed to stress, and consequently, responses belonging the “no” category were classified as non-stressed.

#### Work performance

The outcome measurement at follow-up regarding self-rated *work performance* was assessed by the question “Have your work performance changed during the preceding 12 months?” The response alternatives were (a) “No”, (b) “Yes, improved” and (c) Yes, decreased”. This question has been frequently used in similar studies for measuring self-rated work performance (Boström et al. 2008; Hagberg et al. 2007).

#### Work ability

*Work ability* was assessed at follow-up by a single item from the work ability index (WAI) asking for the current work ability compared with lifetime best, with a possible score ranging from 0 (completely unable to work) to 10 (work ability at its best). This single item WAI has been frequently used in clinical practice and research (Johansson et al. 2011; Sluiter and Frings-Dresen 2008) and has recently been validated by Åhlström and co-workers (Åhlström et al. 2010). The response alternatives were dichotomised according to the recommendation by Åhlström et al., where responses ranging from 0 to 8 were considered indicative of reduced work ability, and responses ranging from 9 to 10 were regarded indicative of good work ability.

### Statistical analysis

Descriptive statistics are given in terms of frequencies and percentages. The outcome measures were dichotomised (decreased work performance (yes or no); and reduced work ability (yes/no) and relations of these outcome variables to the stress and pain variables (exposure variables) were analysed by means of the log binomial model, which is a generalized linear model with a logarithmic link function and binomial distribution function. This is the recommended method for adjusted risk ratio (RR) estimation for common outcomes (prevalence of outcome >10 %) as the odds ratios from logistic regression can overestimate the relative risk under the above mentioned circumstances (Deddens et al. 2004; McNutt et al. 2003; Skov et al. 1998). Based on prior knowledge (scientific and clinical), age (dichotomised into groups ≤45 or >45), gender and physical activity levels (Saltin 1968) were evaluated as possible confounders following the criteria for a confounding factor by Rothman et al. (2008). Finally, potential confounders were included in the model if the change between adjusted and crude RR for the exposure variables was at least 10 % (Hosmer 2000; Rothman et al. 2008). Only the final models are shown in the results.

## Results

Women accounted for four out of five participants, which well mirrors the situation in Swedish health care (Table 1). Twenty-six percent (*n* = 197) reported frequent musculoskeletal pain, and 21 % (*n* = 154) had experienced long-lasting stress at baseline. Decreased work performance at follow-up was reported by 9 % (*n* = 66) and reduced work ability by 34 % (*n* = 246) among those who at baseline reported good work ability and no decrease in work performance.

**Table 1:** Characteristics of the study population at baseline

Characteristics	Distribution % (*n*)
Gender	
Men	20 (151)
Women	80 (595)
Age	
−44	38 (283)
45+	62 (463)
Physical activity	
Sedentary	8 (60)
LPA	51 (381)
MVPA	41 (305)
Stress	
No	79 (589)
Yes	21 (157)
Pain	
No-infrequent	74 (549)
Frequent	26 (197)
Stress/pain	
No/no-infrequent	61 (452)
No/frequent	18 (137)
Yes/no-infrequent	13 (97)
Yes/frequent	8 (60)

Distribution between categories in percent (%) and numbers (*n*)

Participants with complete data for the analyses of work performance (*N* = 746)

*LPA* light physical activity, *MVPA* moderate to vigorous physical activity

Workers who at baseline were categorized as having frequent pain had a higher risk for reporting reduced work ability at follow-up compared to workers without such pain (Table 2). The result was similar to the outcome work performance. Stress was not clearly related to any of the outcomes, although the increased risk estimate for reduced work ability showed a trend towards an association (95 % CI 1.00–1.58). Age was included as a possible confounder in the models for decreased work performance, but not in the models for work ability since it did not change the risk estimates for neither pain nor stress. Gender and physical activity were not associated with either outcome and therefore omitted from the final analyses.

**Table 2:** Percentages, frequencies (*n*) and risk ratios (RR) with 95 % confidence intervals (CI) for stress and musculoskeletal pain in relation to reduced work ability (WAI) and decreased work performance (DWP)

	WAI		DWP	
	% (*n*)	RR (95 % CI)	% (*n*)	RR^a^ (95 % CI)
Stress				
No	32 (184)	1	9 (51)	1
Yes	40 (62)	1.3 (1.00; 1.58)	10 (15)	1.1 (0.63; 1.89)
Pain				
No-infrequent	30 (159)	1	7 (40)	1
Frequent	44 (87)	**1.5 (1.21; 1.81)**	**13 (26)**	**1.5 (1.22; 1.85)**
Stress/pain				
No/no-infrequent	29 (126)	1	8 (34)	1
No/frequent	42 (58)	**1.5 (1.14; 1.86)**	**12 (17)**	**1.5 (1.15; 1.89)**
Yes/no-infrequent	35 (33)	1.2 (0.88; 1.65)	6 (6)	1.2 (0.86; 1.63)
Yes/frequent	49 (29)	**1.7 (1.27; 2.30)**	**15 (9)**	**1.7 (1.28; 2.32)**

No stress and no or infrequent pain constitute reference categories

Bold values indicate statistically significant results (95 % CI does not include 1)

^a^Adjusted for age

Regarding the risk estimates for different combinations of pain and stress, presented in Table 2, the results showed that a combination of frequent pain and perceived long-lasting stress showed the highest risk estimates for reduced work ability and decreased work performance. Frequent pain in combination with no stress significantly increased the risk of reduced work ability and decreased work performance, while a trend towards such a relationship, although not statistically significant, was seen for no/infrequent pain together with perceived stress (Table 2).

## Discussion

The results of the present study have found that frequent musculoskeletal pain is a risk factor for decreased work ability and work performance. These results concur with a cross-sectional study in a non-patient working population, where a strong association between prolonged musculoskeletal pain and reduced work performance was found (Suvinen et al. 2004). Furthermore, these results are in accordance with a study among assistant nurses indicating an association between musculoskeletal well-being and increased work ability (Larsson et al. 2012). These results are also in line with previous longitudinal studies indicating that musculoskeletal pain from at least two locations in the neck and upper extremities and prolonged periods of persistent pain predicts self-reported decrease in productivity (Boström et al. 2008) and that multi-site musculoskeletal pain predicts the development of poor work ability (Neupane et al. 2012). However, contrary results exist. In a large study among a variety of professionals in the UK, no significant association was found between physical health, including musculoskeletal symptoms and self-rated work performance (Donald et al. 2005).

In the present study, perceived stress alone did not increase the risk of reporting decreased work performance or reduced work ability at follow-up. However, a trend towards an influence of long-term stress on work ability was found. Similarly, in the previously mentioned study by Boström et al. (2008), there was a clear trend towards an association between high levels of current stress and self-reported decrease in productivity in the cross-sectional analysis while this relationship, in concordance with the results from our study, no longer existed in the prospective analysis.

Our results indicate that frequent musculoskeletal pain in combination with perceived long-lasting stress at baseline is associated with a decreased work ability and work performance at follow-up. In sum, frequent musculoskeletal pain seems to be directly related to decreased work performance and work ability, on its own, and also in combination with exposure to long-lasting stress, whereas the effects of exposure to long-lasting stress only are less clear. Adverse psychosocial working conditions have been identified as closely connected to musculoskeletal pain in previous studies (Bongers et al. 2002, 2006). There is some research suggesting that such adverse working conditions are related to musculoskeletal pain through their effects on perceived stress, that is, work stressors such as high job demands are hypothesized to cause high job stress, which in turn cause musculoskeletal pain through, for example, an increased muscle tension (Stewart et al. 2003a, b). Potential implications for the interpretation of our results (the absence of a relationship between stress and reduced work ability/work performance, but a clear relationship between musculoskeletal pain and reduced work ability and work performance) may therefore be that participants in this study who report frequent musculoskeletal pain might have been exposed to a higher and more prolonged exposure to work-related stressors and that exposure to a high job stress is more harmful when it is manifested also in physical symptoms. Both clinical experience and the scientific literature in the field indicate that exposure to adverse psychosocial working conditions often first expresses itself as physical sensations (Holte et al. 2003; Wahlstrom et al. 2003) and that these sensations may be the first “signs” of prolonged exposure to stress and sometimes precede more severe stress-related mental conditions like exhaustion disorder/clinical burnout or depression, which often lead to sickness absence. Our findings therefore indicate the possibility that frequent musculoskeletal pain with or without long-standing stress as a contributing cause is associated with decreased work ability and work performance, while the perception of stress, not accompanied by pain (although other physical sensations or symptoms may exist), suggests an earlier and less severe stage in relation to these adverse outcomes.

Work ability has been measured in many different ways in the literature sometimes by using the whole WAI (Ilmarinen 2007) and sometimes by using single questions (van den Berg et al. 2011). Moreover, in some studies, sick leave has been used as a measure of work ability, for example, in terms of not being on long-term sick leave or categorized by the amount of sick leave days in the preceding 12-month period (Lindberg et al. 2006). In this study, we chose to use the single item question included in the WAI that requests the responder to estimate the current perceived work ability compared to his/her best perceived work ability ever. It could be discussed whether using a single item taken out of an established scale context could be justified, but this question have been scrutinized with respect to validity and reliability and found to be both valid and useful in order to assess current status and development of perceived work ability among women on long-term sick leave (Åhlström et al. 2010).

The effects of individual and work-related factors on work ability measured with the WAI have been viewed in a recent review by van den Berg and co-workers, and they conclude that poor work ability is associated, amongst other things, with high mental workload, poor physical work environment and lack of leisure physical activity (van den Berg et al. 2011). The leisure physical activity level was in our study treated as a potential confounder, but was excluded from the final analysis since the level of physical activity was not associated with the outcomes or the exposure variables in our data and thus did not fulfil the criteria of a true confounder (Rothman et al. 2008).

Stress was in our study measured as perceived stress persisting for at least 1 month during the preceding 12 months. Many other studies use only current stress as a measure of stress exposure. With respect to our outcome measurements, work ability and work performance, it is not likely to believe that measuring current stress solely would have any strong impact on our outcome measurements due to the fact that short periods of repeated stress (acute stress) with sufficient recuperation in between is not considered to be related to neither hazardous stress reactions nor with more manifest stress-related disorders (de Kloet et al. 2005; McEwen 1998).

### Strengths and limitations

The strength of this study is above all the longitudinal design which allows us to, although with caution, draw conclusions about causal effects of the exposure to frequent pain and perceived stress on work ability and work performance, and thus strengthen the implication for preventive measures aiming at reducing musculoskeletal pain and perceived stress both on the individual as well as on the organizational level. However, in our study, we have not investigated the magnitude of the impact of frequent musculoskeletal pain and perceived stress in relation to other risk factors regarding influence on work ability and work performance, since this was not the aim of the study. Thus, unknown risk factors might have been concurrently present during the follow-up period.

Articles investigating the impact of stress and work environment on productivity (work performance) and work ability have sometimes been criticized for deficits in data collection, for instance not having enough variability in the investigated target groups, and including small samples (Donald et al. 2005). In our study, we have tried to address these issues by using a fairly big sample size (*n* = 770) with different professions included (for example, paramedics, assistant nurses, nurses, physicians, cleaners, administrators, engineers and managers). However, employees from only one organization were included in this study, which could be a limitation, but, on the other hand, the variety of work tasks and work environments within this organization with workplaces spread over a larger geographical region might compensate for this shortcoming.

## Conclusions

A recent review has concluded that, among other things, poor musculoskeletal capacity and high mental work demands are associated with poor work ability (van den Berg et al. 2009). Our study contributes by adding frequent musculoskeletal pain, especially in combination with perceived long-standing stress, to the list of factors negatively influencing work performance and work ability. We suggest that the practical implication from this study is that proactive workplace interventions, especially in human service organizations, in order to maintain high work performance and good work ability should include measures to promote good musculoskeletal well-being for the employees as well as measures, both individual and organizational, to minimize the risk of persistent stress reactions.
